# *Translation of the Report Lately Made to the Class of Mathematical and Physical Sciences, of the National Institute of France, in Relation to the Prize Founded by the First Consul, Subscribed by the Names*, Laplace, Halle, Coulomb, Hauy, *and* Biot, *Reporter*

**Published:** 1804-06-01

**Authors:** 


*Translation of the Report lately made to the Class of Mathematical and Physical Sciences, of the National Institute of France, in relation to the Prize founded by the First Consul, subscribed by the Names,* LAPLACE, HALLE, COULOMB, HAUY, *and* BIOT, *Reporter.*

THE National Institute, which has taken an active part in the great discoveries with which the theory of Electricity has been lately enriched, feels, in all its extent, the importance of the subject indicated by the First Consul. Of all the physical forces to which the bodies of Nature are subjected, electricity appears to be that which manifests its its influence the most frequently. It not only acts on inorganic bodies, which it modifies or decomposes, but organized bodies themselves experience the most astonishing effects from it. That which was, for the antients, only a simple result of some attractive properties, is become, for modern philosophers, the source of the most brilliant discoveries.

We may divide the history of electricity into two periods, which are distinguished, as much by the nature of the results, as by that of the apparatus employed to obtain them. In the one the electric influence is produced by the rubbing of glass or resinous matters; in the other, electricity is put in motion by the simple contact of bodies between themselves. We should refer to the first of these two epochs, the distinction of the two species of electricity, resinous and vitreous, the analysis of the Leyden bottle, the explication of the thunder-storm, the invention of *paratonneres*, (Franklyn's iron rods, &c.) and the exact determination of the laws, according to which the repulsive force of the electric matter varies with the distance. The second comprehends the discovery of the muscular contractions, excited by the contact of metals, the explication of these phenomena by the movement of metallic electricity; and, lastly, the formation of the electric column, its analysis, and its different properties. Volta has performed in this second period, what Franklyn did in the first.

The sciences are now so connected among themselves, that whatever serves to perfect the one, at the same time advances the others. Under this point of view, Galvanism will form a memorable epoch in their history; for there are few discoveries which have given to Physics and Chemistry so many new facts, and so different from what were known before. Already has the *ensemble* of these facts been referred to a general cause, which is the movement of electricity; it remains to determine with precision the circumstances which accompany them, to follow the numerous applications which they offer, and to discover the general laws which are perhaps included in them.

The greater part of the chemical effects exhibited by the new apparatus have not received explications completely satisfactory, and it is so much the more important to know them well, as they furnish to chemistry, means sufficiently powerful to decompose the most intimate combinations. It is equally interesting to examine whether the electric properties that certain minerals acquire in their variations of temperature, do not depend on a disposition of their elements elements, analogous to that which constitutes the column of Volta. And lastly, it is desirable that the theory of electricity, augmented by these new phenomena, should be compleatly submitted to calculation in a general, direct, and rigorous manner; and the steps that have been already taken in this career have proved that this difficult subject demands the sagacity of ingenious Physics, and the succours of the most profound Analectics.

But it is, above all, in their application to animal economy, that it highly imports us to consider the galvanic apparatus. It is already known that metals are not the only substances, the contact of which determines the movement of electricity. This property is common to them with certain liquids; and it is probable that it extends, with different modifications, to all the bodies of Nature. Do not the phenomena which the torpedo and other electrical fishes offer, depend upon an analogous action, which may be exercised between the different parts of their organization? And does not that action exist, with a degree of intensity less sensible, but not less real, in a number of animals, much more considerable than has been generally believed? An accurate analysis of these effects, a complete explication of the mechanism which determines them, and the aggregation of those which the column of Volta presents, would give, perhaps, a key to the most important secrets of animal physics. In thus considering the *ensemble* of these phenomena, we can presage the possibility of a great discovery, which, by developing a new law of Nature, would refer them to one single cause, and connect them with those which the movement of electricity furnishes us with, in minerals.

These considerations have doubtless had their due influence on the class; and if it has not proposed a prize for the improvement of this part of physics, it is because the extent of the subject seeming to demand more than one *concours*, it could not devote to that alone, the encouragements which it owes, in general, to all the branches of useful knowledge. However, each one of its members, and all the learned of Europe, ought seriously to wish, that the researches of Naturalists may be directed towards this important end, and they should congratulate themselves on seeing their wishes now fulfilled in the most compleat manner.

To answer the intentions of the First Consul, and to give to this *concours* all the solemnity that the importance of the object, the nature of the prize, and the character of him that has founded it, require, the Commission unanimously mously proposes the following project: The Class of Mathematical and Physical Sciences of the National Institute, opens the general *concours* demanded by the First Consul. All the learned of Europe, including the members resident and associate of the National Institute, are invited to the *concours*. The Class does not expect that the Memoirs should be directly addressed to it. It will crown every year, the author of the best experiments that shall come to its knowledge, and which shall have accelerated the progress of science. The grand prize will be given to the person whose discoveries in the history of Electricity and of Galvanism, will form an æra, or a memorable epoch. The present report, including the letter of the First Consul, will be printed, and will serve for a program. Done at the National Institute, 11 Messidor, year 10.

